# HYPERTENSION, AGE AND SEX INFLUENCE REGIONAL CEREBRAL BLOOD FLOW IN OLDER ADULTS

**DOI:** 10.1093/geroni/igae098.3664

**Published:** 2024-12-31

**Authors:** Jasroop Miglani, Nathanial Ashford, Kristen Farris, Swati Rane Levendovszky, Angela Hanson

**Affiliations:** University of Washington School of Medicine, Seattle, Washington, United States; University of Washington School of Medicine, Seattle, Washington, United States; University of Washington School of Medicine, Seattle, Washington, United States; University of Washington School of Medicine, Seattle, Washington, United States; University of Washington, Kenmore, Washington, United States

## Abstract

**Background:**

Reduced cerebral blood flow (CBF) is a potential early biomarker for dementia and older adults with hypertension (HTN) are at increased risk of accelerated cognitive decline. This study explored the influence of HTN, age, and sex on CBF following lipid ingestion.

**Methods:**

36 older adults (age 66.9 ± 7.2, 44% hypertensive, 67% women) underwent arterial spin labeling 3D pCASL MRI to measure CBF at baseline (t0) and 2 hours after drinking heavy cream (t2). Regions examined: total cerebrum (tCBF), angular gyrus (AGBF), hippocampus (HBF), posterior cingulate (PCBF), and temporal lobe (TBF). Statistics included Pearson correlation in SAS and generalized linear modeling in R.

**Results:**

Age negatively correlated with tCBF at t0 (CC -0.33, p=0.05) which was magnified after lipid ingestion (CC -0.41, p=0.01); similar findings noted for other regions. Men had lower tCBF than women at t0 (Coefficient -8.918, p=0.003) and t2 (Coefficient -11.3, p< 0.001); pattern held for all regions examined. When covariates were added to the model, men were more likely to be hypertensive which partially explained this finding; however, the sex effect remained significant after controlling for factors known to affect CBF (age, heart rate, hematocrit, SBP).

**Conclusion:**

Age correlated with lower CBF both fasting and after lipid feeding. Men had lower CBF than women which was only partially explained by HTN; a finding that has been reported in the literature. Further investigation into how various demographic variables influence CBF is crucial in order to utilize CBF as an early biomarker in clinical trials.

